# Outcomes and Predictors of Mortality in Hospitalized Frail Patients Undergoing Percutaneous Coronary Intervention

**DOI:** 10.7759/cureus.5399

**Published:** 2019-08-16

**Authors:** Rupak Desai, Alok R Amraotkar, Melissa G Amraotkar, Samarthkumar Thakkar, Hee Kong Fong, Yash Varma, Nanush Damarlapally, Rajkumar P Doshi, Kishorbhai Gangani

**Affiliations:** 1 Cardiology, Atlanta Veterans Affairs Medical Center, Decatur, USA; 2 Cardiovascular Medicine, University of Louisville School of Medicine, Louisville, USA; 3 Nursing Education, University of Louisville School of Nursing, Louisville, USA; 4 Internal Medicine, Rochester General Hospital, Rochester, USA; 5 Cardiovascular Medicine, University of California Davis Medical Center, Sacramento, USA; 6 Internal Medicine, Government Medical College, Bhavnagar, IND; 7 Health Sciences, Coleman College of Health Sciences, Houston, USA; 8 Internal Medicine, University of Nevada, Reno School of Medicine, Reno, USA; 9 Internal Medicine, Texas Health Arlington Memorial Hospital, Arlington, USA

**Keywords:** frailty, all-cause mortality, cardiovascular outcomes

## Abstract

Objective

To study the impact of frailty on inpatient outcomes among patients undergoing percutaneous coronary intervention (PCI).

Methods

The National Inpatient Sample data of all PCI-related hospitalizations throughout the United States (US) from 2010 through 2014 was utilized. Patients were divided into two groups: frailty and no-frailty. International Classification of Diseases, Ninth Revision, Clinical Modification (ICD-9-CM) codes were used to stratify groups and outcomes. In order to address the substantial difference in the total number of valid observations between the two groups, a propensity-matched analysis was performed at a 1:1 ratio and caliper width of 0.01.

Results

A total of 2,612,661 PCI-related hospitalizations throughout the US from 2010 through 2014 were identified, out of which 16,517 admissions (0.6%) had coexisting frailty. Only 1:1 propensity-matched data was utilized for the study. Propensity-matched frailty group (n=14,717) as compared to no-frailty (n=14,755) was frequently older, white, and Medicare enrollee (p<0.05). The frailty group had significantly higher rates of comorbidities and complications (p<0.05). All-cause in-hospital mortality was higher in the no-frailty group (p<0.05). Age, white race, non-elective admission, urban hospitals, and comorbidities predicted in-hospital mortality in frailty group (p<0.05). Rheumatoid arthritis, depression, hypertension, obesity, dyslipidemia, and history of previous PCI decreased odds of in-hospital mortality in frailty group (p<0.05). Frailty group had prolonged hospital stay and higher hospital charges (p<0.05).

Conclusions

Frailty has a significant effect on PCI-related outcomes. We present a previously unknown protective effect of cardiovascular disease risk factors and other health risk factors on frail patients undergoing PCI. Frailty’s inclusion in risk stratification will help in predicting the post-procedure complications and improve resource utilization.

## Introduction

Frailty is described as the presence of three out of the following five criteria of compromised energetics: low grip strength, low energy, slowed walking speed, low physical activity, or unintentional weight loss [1]. Although not a gold standard, there is a broad acceptance of the above criteria in the clinical arena. Frailty significantly affects mortality and disability outcomes [1]. Despite its prognostic importance, frailty is not formally evaluated in clinical practice. Frailty is a known risk factor for cardiovascular diseases (CVD) and their combined deleterious outcomes [2,3]. However, there is limited data on the impact of frailty on CVD outcomes, especially in patients undergoing percutaneous coronary intervention (PCI). PCI is routinely utilized to treat patients with acute or non-acute coronary artery disease effectively, and elderly patients are the most commonly treated demographic population [4]. Given the physical and metabolic changes in elderly patients, there is a high chance of encountering frailty in them [5].

The intersection of frailty in cardiac and extra-cardiac comorbidities is critical in understanding the short- and long-term impact of frailty on CVD outcomes. Therefore, we propose a study to investigate the impact of frailty in post-PCI patients. The primary aim of this study is to investigate outcomes (mortality and other complications) and predictors of mortality in hospitalized frail patients undergoing PCI. The second aim of this study is to investigate the impact of frailty on hospital stay.

## Materials and methods

Population and design

The National Inpatient Sample (NIS) is the largest publicly accessible all-payer inpatient database in the United States (US) and is sponsored by the Agency for Healthcare Research and Quality (AHRQ) as a part of the Healthcare Cost and Utilization Project (HCUP) [6]. NIS data from 2010 through 2014 was used for this study. The discharge weights were applied to attain the national estimates, which minimizes the margin of error representing over 95% of the US population. Adult patients (age 18 and over) undergoing PCI were identified via International Classification of Diseases, Ninth Revision, Clinical Modification (ICD-9-CM) procedural codes 00.66, 36.01, 36.02, 36.05, 36.06, 36.07, and 17.55. Other diagnoses, comorbidities, complications, and discharges were identified using ICD-9-CM codes as detailed in our previous studies. The study population was divided into two groups: “frailty” and a control group - “no frailty”. “Frailty” was defined via ICD-9-CM 797, 799.3 & 783.7 as done previously [7,8]. The study did not require the ethics board approval as NIS is obtainable publicly and does not reveal patients’ identifiers.

Primary and secondary outcomes

There were two primary outcomes in the study. The first primary outcome was defined as inpatient outcomes (mortality, other complications). The second primary outcome was defined as predictors of in-hospital mortality (multivariable). The secondary outcome was defined as impact on hospital stay (discharge, length of stay (LOS), and hospital charges). The discharge of patients was further categorized into four sub-categories: routine, transfer to short-term hospital, nursing/other facilities (skilled or intermediate), and other transfers.

Statistical analysis

**Figure 1:**
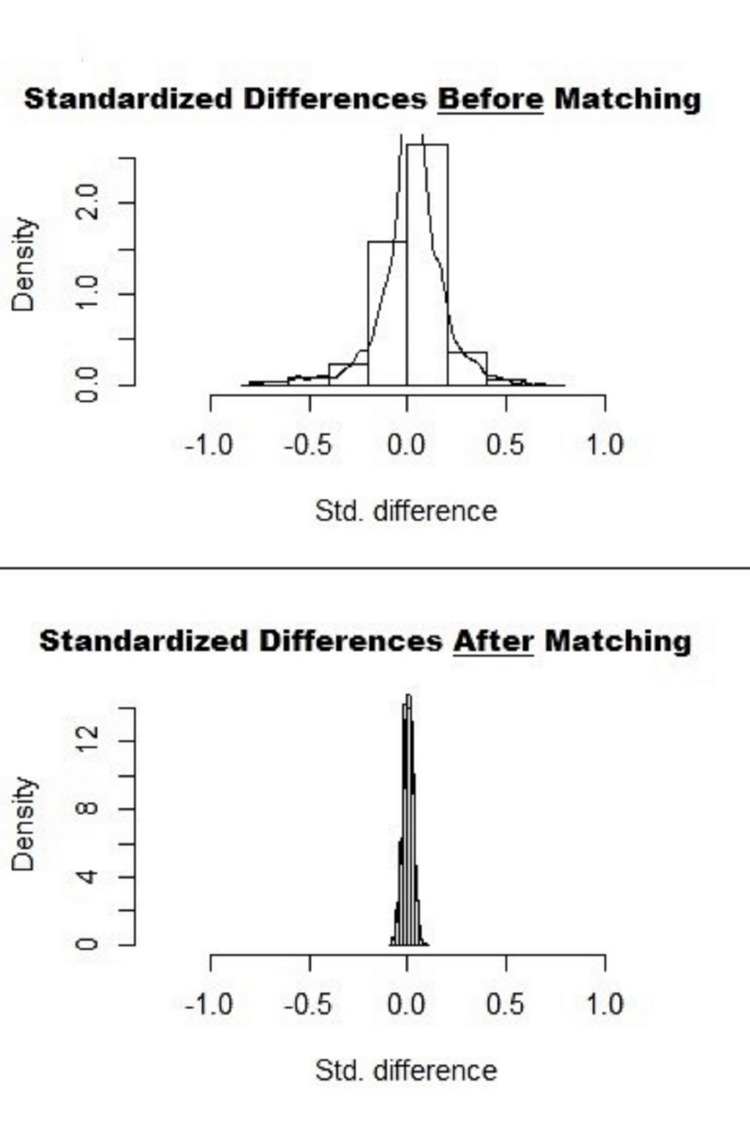
Standardized Differences in Data Before and After 1:1 Propensity Matching Std. Difference = Standardized Differences

A two-tailed p<0.05 was considered a threshold for clinical significance. Due to a substantial difference in the total number of valid observations between the two groups, a propensity-matched analysis was performed with a ratio of 1:1 without replacement using a caliper width of 0.01. The absolute standardized difference of <10% was obtained for most variables before and after propensity matching (Figure 1).

Data was matched with all baseline characteristics, comorbidities, and hospital characteristics. Only 1:1 propensity-matched data were utilized to assess primary and secondary outcomes. Chi-square test and independent-sample t-test were performed to compare the baseline characteristics. Outcomes and predictors were adjusted for age, sex, race, median income, payer status, hospital characteristics, and relevant comorbidities. Odds ratios (OR) and 95% confidence intervals (CI) were calculated for mortality predictors. International Business Machines Corporation (IBM) Statistical Package for the Social Sciences (SPSS) v22.0 (IBM Corp., Armonk, NY) was utilized to perform the analyses.

## Results

General cohort characteristics

A total of 2,612,661 PCI-related hospitalizations throughout the US from 2010 through 2014 were identified, out of which 16,517 admissions (0.6%) had coexisting frailty (Tables 1, 2). Only 1:1 propensity-matched data was utilized for this study. The unmatched original data are detailed in Tables 1, 2. After a 1:1 propensity-matching, there were 14,717 patients in the frailty group and 14,755 patients in the no-frailty group (Tables 3, 4). 

**Table 1:** General Characteristics of No-Frailty versus Frailty Patients Undergoing Percutaneous Coronary Intervention Without Any Propensity Matching *Derived from https://www.hcup-us.ahrq.gov/db/vars/zipinc_qrtl/nisnote.jsp SD=Standard Deviation p-value <0.05 indicates statistical significance

Characteristics	No-Frailty (n=2,596,144)	Frailty (n=16,517)	p-value
Age Mean±SD (Yrs)	64 ± 12	75 ± 11	<0.001
Sex			<0.001
Male (%)	67.1	49.5	
Female (%)	32.9	50.5	
Race			<0.001
White (%)	76.7	78.3	
African American (%)	9.2	9.5	
Hispanic (%)	7.3	7.3	
Asian Pacific Islander (%)	2.4	2.0	
Native American (%)	0.6	0.4	
Others	3.7	2.5	
Type Of Admission			<0.001
Non-Elective (%)	82.2	90.0	
Elective (%)	17.8	10.0	
Admission Day			<0.001
Weekday (%)	79.6	76.8	
Weekend (%)	20.4	23.2	
Median Household Income^*^			<0.001
0-25^th^ (%)	28.7	32.9	
26-50^th^ (%)	27.0	28.2	
51-75^th^ (%)	24.4	22.4	
76-100^th^ (%)	19.9	16.4	
Primary Expected Payer			<0.001
Medicare (%)	51.4	80.7	
Medicaid (%)	7.2	4.3	
Private (%)	31.6	11.8	
Self-Pay/No Charge/Other (%)	9.9	3.2	
Location/Teaching Status Of Hospital			<0.001
Rural (%)	6.4	6.9	
Urban Non-Teaching (%)	37.3	40.8	
Urban Teaching (%)	56.3	52.3	
Region Of Hospital			<0.001
Northeast (%)	18.5	3.9	
Midwest (%)	25.3	32.1	
South (%)	39.3	48.7	
West (%)	16.9	15.3	

**Table 2:** Comorbidities in No-Frailty versus Frailty Patients Undergoing Percutaneous Coronary Intervention Without Any Propensity Matching p-value <0.05 indicates statistical significance

Characteristics	No-Frailty (n=2,596,144)	Frailty (n=16,517)	p-value
Alcohol Abuse (%)	2.6	2.9	0.012
Chronic Pulmonary Disease (%)	17.3	33.0	<0.001
Coagulopathy (%)	3.2	8.6	<0.001
Congestive Heart Failure (%)	1.3	6.9	<0.001
Depression (%)	6.9	12.7	<0.001
Hypertension (%)	73.6	76.6	<0.001
Diabetes, With Chronic Complications (%)	5.3	11.7	<0.001
Diabetes, Uncomplicated (%)	31.2	34.1	<0.001
Dyslipidemia (%)	70.7	62.2	<0.001
Drug Abuse (%)	2.0	1.6	0.002
Fluid And Electrolyte Disorders (%)	13.2	41.9	<0.001
Valvular Heart Disease (%)	0.4	2.1	<0.001
Obesity (%)	15.6	17.8	<0.001
Peripheral Vascular Disorders (%)	11.1	20.2	<0.001
Renal Failure (%)	13.7	33.7	<0.001
Smoking (%)	41.8	31.1	<0.001

**Table 3:** General Characteristics of No-Frailty versus Frailty Patients Undergoing Percutaneous Coronary Intervention From 1:1 Propensity Matched Data *Derived from https://www.hcup-us.ahrq.gov/db/vars/zipinc_qrtl/nisnote.jsp SD=Standard Deviation p-value <0.05 indicates statistical significance

Characteristics	No Frailty (n=14,755)	Frailty (n=14,717)	p-value
Age Mean±SD (Years)	73 ± 11	75 ± 11	<0.001
Sex			0.99
Male (%)	49.6	49.6	
Female (%)	50.4	50.4	
Race			<0.001
White (%)	79.5	78.9	
African American (%)	8.9	8.8	
Hispanic (%)	7.1	7.5	
Asian Pacific Islander (%)	1.9	2.0	
Native American (%)	0.1	0.4	
Others	2.3	2.4	
Type Of Admission			0.83
Non-Elective (%)	90.0	89.9	
Elective (%)	10.0	10.1	
Admission Day			0.10
Weekday (%)	77.9	77.1	
Weekend (%)	22.1	22.9	
Median Household Income*			0.54
0-25^th^ (%)	33.6	32.9	
26-50^th^ (%)	27.3	27.9	
51-75^th^ (%)	22.4	22.5	
76-100^th^ (%)	16.8	16.7	
Primary Expected Payer			<0.001
Medicare (%)	82.3	80.7	
Medicaid (%)	3.9	4.3	
Private (%)	11.6	11.8	
Self-Pay/No Charge/Other (%)	2.3	3.2	
Location/Teaching Status Of Hospital			0.47
Rural (%)	6.3	6.6	
Urban Non-Teaching (%)	42.5	42.1	
Urban Teaching (%)	51.3	51.3	
Region Of Hospital			0.09
Northeast (%)	3.8	4.2	
Midwest (%)	27.4	27.9	
South (%)	52.5	52.0	
West (%)	16.4	15.9	

**Table 4:** Comorbidities in No-Frailty versus Frailty Patients Undergoing Percutaneous Coronary Intervention From 1:1 Propensity Matched Data p-value <0.05 indicates statistical significance

Comorbidities	No Frailty (n=14,755)	Frailty (n=14,717)	p-value
Alcohol Abuse (%)	2.8	3.0	0.51
Chronic Pulmonary Disease (%)	32.7	32.4	0.61
Coagulopathy (%)	7.9	8.5	0.041
Congestive Heart Failure (%)	5.8	7.0	<0.001
Depression (%)	12.2	12.6	0.33
Hypertension (%)	76.4	76.4	0.98
Diabetes, With Chronic Complications (%)	12.4	11.8	0.65
Diabetes, Uncomplicated (%)	33.8	34.1	0.12
Dyslipidemia (%)	63.4	62.2	0.037
Drug Abuse (%)	1.9	1.7	0.21
Fluid And Electrolyte Disorders (%)	41.3	42.4	0.07
Valvular Heart Disease (%)	1.5	2.3	<0.001
Obesity (%)	18.2	17.9	0.50
Peripheral Vascular Disorders (%)	19.6	20.2	0.23
Renal Failure (%)	34.0	33.7	0.57
Smoking (%)	30.7	31.2	0.34

Propensity matched cohort characteristics

General cohort characteristics of 1:1 propensity-matched cohort are presented in Table 3. The frailty group was frequently older (mean age 75+11 years), white (78.9%), and Medicare enrollees (80.7%). Cohort comorbidities are presented in Table 4. The frailty group had significantly higher rates of coagulopathy (8.5% vs 7.9%, p=0.041), congestive heart failure (7% vs 5.8%, p<0.001), and valvular heart disease (2.3% vs 1.5%, p<0.001) as compared to no-frailty group (Table 2). However, dyslipidemia was lower in the frailty group (62.2% vs. 63.4%, p=0.037) as compared to the no-frailty group (Table 4).

First primary outcome: inpatient outcomes in no-frailty and frailty groups

**Figure 2:**
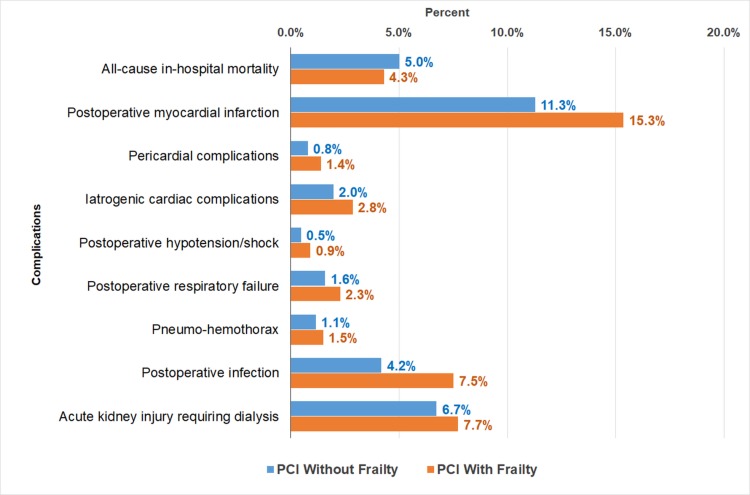
Inpatient Outcomes in Frailty versus No-Frailty Patients Undergoing Percutaneous Coronary Intervention From 1:1 Propensity Matched Data PCI = Percutaneous Coronary Intervention p-value <0.05 indicates statistical significance

The propensity-matched inpatient outcomes (mortality and other complications) are presented in Figure 2. The all-cause inpatient mortality was significantly higher in no-frailty group (5% vs 4.3%). Postoperative myocardial infarction (15.3% vs 11.3%), pericardial complications (1.4% vs 0.8%), iatrogenic cardiac complications (2.8% vs 2%), postoperative hypotension/shock (0.9% vs 0.5%), postoperative respiratory failure (2.3% vs 1.6%), pneumo-hemothorax (1.5% vs 1.1%), postoperative infection (7.5% vs 4.2%), and acute kidney injury during dialysis (7.7% vs 6.7%) were all significantly higher in the frailty group (all p<0.05) (Figure 2).

Second primary outcome: predictors of inpatient mortality in frailty group

**Figure 3:**
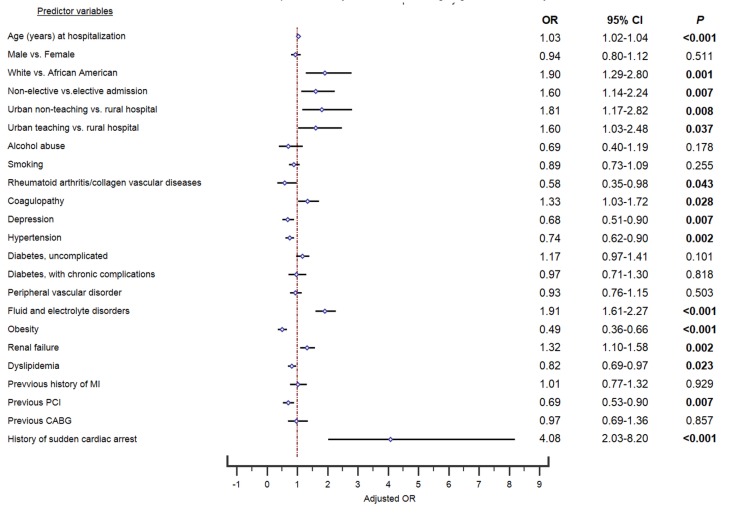
Multivariable Predictors of Inpatient Mortality in Frailty Patients From 1:1 Propensity Matched Data CABG = Coronary Artery Bypass Graft, CI = Confidence Interval, OR = Odds Ratio, MI = Myocardial Infarction, P = p-value, PCI = Percutaneous Coronary Intervention p-value <0.05 indicates statistical significance

The propensity-matched multivariable logistic regression analysis for predictors of in-hospital mortality are detailed in Figure 3. The odds of inpatient mortality in the frailty group were increased with age (OR=1.03, 95% CI: 1.02-1.04, p<0.001), white race (OR=1.90, 95% CI: 1.29-2.80, p<0.001), non-elective admission (OR=1.60, 95% CI: 1.14-2.24, p=0.007), treatment at urban non-teaching hospital (OR=1.81, 95% CI: 1.17-2.82, p=0.008) or urban teaching hospital (OR=1.60, 95% CI: 1.03-2.48, p=0.037). The odds of in-hospital mortality were increased in the frailty group with the presence of following comorbidities: coagulopathy (OR=1.33, 95% CI: 1.03-1.72, p=0.028), fluid and electrolyte disorders (OR=1.91, 95% CI: 1.61-2.27, p<0.001), renal failure (OR=1.32, 95% CI: 1.10-1.58, p=0.002), and history of sudden cardiac arrest (OR=4.08, 95% CI: 2.03-8.20, p<0.001).

The odds of in-hospital mortality were decreased in the frailty group with the presence of following comorbidities: rheumatoid arthritis/collagen vascular diseases (OR=0.58, 95% CI: 0.35-0.98, p=0.043), depression (OR=0.68, 95% CI: 0.51-0.90, p=0.007), hypertension (OR=0.74, 95% CI: 0.62-0.90, p=0.002), obesity (OR=0.49, 95% CI: 0.36-0.66, p<0.001), dyslipidemia (OR=0.82, 95% CI: 0.69-0.97, p=0.023), and history of previous PCI (OR=0.69, 95% CI: 0.53-0.90, p=0.007).

Secondary outcome: impact on hospital stay (discharge, LOS, and hospital charges)

Routine discharges (65.2% vs 30.2%, p<0.001) and transfers to short-term hospital (1.4% vs 0.9%, p<0.001) were significantly higher in the no-frailty group. Whereas, transfers to nursing/other facilities (21.9% vs. 14%, p<0.001) and all other transfers (42.2% vs. 14.3%, p<0.001) were significantly higher in the frailty group. Frailty group had prolonged mean LOS (9.5+7.8 vs 5.9+6.6 days, p<0.001) and higher mean hospital charges ($141,532 vs $108,468, p<0.001).

## Discussion

In this retrospective analysis of PCI-related hospitalizations in frail and non-frail patients, we provide a first-ever attempt at quantifying the impact of frailty on PCIs performed across the US between 2010 and 2014. There were three major novel findings with important clinical implications in this study. First, with the exception of all-cause in-hospital mortality, a majority of in-hospital complications were significantly associated with frailty in patients undergoing PCI. Second, there was a >50% chance of in-hospital mortality in frail patients if they were-white, non-electively admitted, treated at an urban hospital, had fluid/electrolyte disorders, or a history of sudden cardiac arrest. A history of sudden cardiac arrest alone increased the chance of in-hospital mortality in frail patients by >300%. Third, the chances of in-hospital mortality in frail patients decreased by >20% if patients had coexisting rheumatoid arthritis/collagen vascular diseases, depression, hypertension, obesity, dyslipidemia, or a history of previous PCI. Additionally, the cost of treatment and length of stay were both significantly higher in frail patients as compared to non-frail patients.

There is a high incidence of post-procedural complications in older patients undergoing cardiovascular interventions, which may be due to a higher number of comorbidities, recurrent multivessel diseases, and or disparities in resource utilization [9-12]. Moreover, frailty reduces the abilities of stress management, thereby accentuating patients’ suffering [13]. In our study, the post-PCI all-cause in-hospital mortality rate was higher in non-frail patients than frail patients, which is contradictory to the findings of previous studies [9-11]. Our study presents a novel finding that a history of previous PCI decreased the odds of mortality in frail patients, thereby highlighting a protective effect of a history of PCI for this population. One of the reasons for such contradictory findings could be decreased CVD burden at the time of the study, secondary to successful disease management in the preceding PCIs.

In stark contrast with expected clinical outcomes, pre-existing rheumatoid arthritis, depression, hypertension, obesity, and dyslipidemia a were all associated with reduced odds of inpatient mortality in frail patients undergoing PCI. These findings may have considerably influenced the observed decrease in overall in-hospital mortality in frail patients in our study. Consistent with prior reports of the “Obesity Paradox”, frail patients with obesity in our study were protected (by almost 50%) against post-PCI in-hospital mortality. The protective effect of obesity in critically ill patients has been revealed in multiple recent studies [14,15]. In a recently published study, Leistner et al. found abnormally low BMI in elderly patients associated with increased mortality [15]. Similarly, both Lavie et al. (2014) and Kapoor et al. (2010), have found an association of frailty and cachexia with poorer prognosis as compared to being obese in patients with heart failure [14,16]. The neutralization of inflammatory signals by adipose tissue via the production of soluble receptors may be a key factor in this phenomenon [17]. Lipid-lowering treatment has been previously shown not to affect mortality in the elderly population, and low total cholesterol is in-fact associated with higher mortality [18]. This can explain the protective effect of dyslipidemia on in-hospital mortality in frail patients in our study. 

Although smoking remains a widely reported CVD risk factor for worse outcomes, it did not play a role in predicting short-term outcomes for frail patients in our study. The rising burden of endocrine disorders, including diabetes mellitus and hypothyroidism among the US population, is known to play a role in PCI outcomes [19]. However, in our study, we did not observe a correlation between diabetes or hypothyroidism and in-hospital negative outcomes in frail patients.

Between varieties of different hospital admission causes, the black race has historically been associated with lower resource utilization and higher in-hospital mortality [10-12,20-22]. However, among frail patients in our study, the white race was the strongest racial predictor of inpatient mortality. This finding could be due to the sociodemographic profile of the study sample with a majority of the cohort (with or without frailty) being white.

CVD is the leading cause of death in the US [23]. Paradoxically, hypertension, obesity, and dyslipidemia, the three major CVD risk factors, were found to decrease the odds of in-hospital mortality in frail patients in our study. These findings warrant further investigations regarding the role of these CVD risk factors in predicting short-term or long-term outcomes in frail patients undergoing revascularization.

Limitations

As with any administrative database study, this study has a few potential limitations. Under/over-reporting of frailty could be a limitation considering our methodology involved ICD-9-CM codes to extract frail patients with a possibility of administrative coding errors. The database does not provide follow-up data. In addition, we could not grade the severity of frailty through indices.

## Conclusions

Frailty has a significant effect on PCI-related outcomes and should be included as a fundamental clinical condition while evaluating risk factors for inpatient outcomes and mortality benefits surrounding PCI. This study presents a previously unknown protective effect of CVD risk factors and other health risk factors on frail patients undergoing PCI procedure. Specifically designed studies are warranted to further investigate the paradoxical findings of expected clinical outcomes in this study, which will add vital value for managing this growing patient population. 
